# Optimizing nicardipine dosage for effective control of pituitrin-induced hypertension in laparoscopic myomectomy undergoing total intravenous anesthesia

**DOI:** 10.1186/s12871-024-02521-w

**Published:** 2024-04-23

**Authors:** Chen Wang, Xiaoli Zhao, Yunyun Chen, Jianhua Xia, Xixue Zhang, Tingting Wang

**Affiliations:** 1 Department of Anaesthesia, Seventh People’s Hospital of Shanghai Universityof Traditional Chinese Medicine, Shanghai, 200137 China; 2 Department of Anaesthesia, Changning Maternity and Infant Health Hospital, Shanghai, 200050 China; 3 Department of Anaesthesia, Shanghai Pudong New Area People´s Hospital, Shanghai, 201299 China; 4 Department of Anaesthesia, Huadong Hospital affiliated to Fudan University, No 221, West Yan’an Road, Shanghai, 200040 China

**Keywords:** Uterine fibroids, Laparoscopic myomectomy, Pituitrin, Nicardipine, Hemodynamic response, Up-and-down methods, ED50, ED95

## Abstract

**Background:**

This study aimed to determine the median effective dose (ED50) and 95% effective dose (ED95) of nicardipine for treating pituitrin-induced hypertension during laparoscopic myomectomy, providing guidance for the management of intraoperative blood pressure in such patients.

**Methods:**

Among the initial 40 participants assessed, 24 underwent elective laparoscopic myomectomy. A sequential up-and-down method was employed to ascertain the ED50 of nicardipine based on its antihypertensive efficacy. Nicardipine was initially administered at 6 µg/kg following the diagnosis of pituitrin-induced hypertension in the first patient. Dosing adjustments were made to achieve the desired antihypertensive effect, restoring systolic blood pressure and heart rate to within ± 20% of baseline within 120 s. The dosing increment or reduction was set at 0.5 µg/kg for effective or ineffective responses, respectively. The ED50 and ED95 of nicardipine were calculated using Probit regression by Maximum Likelihood Estimation (MLE) to establish dose-response curves and confidence intervals.

**Results:**

24 patients were included for analysis finally. The ED50 and ED95 of nicardipine for blood pressure control after pituitrin injection were determined. The study found that the ED50 of nicardipine for treating pituitrin-induced hypertension was 4.839 µg/kg (95% CI: 4.569–5.099 µg/kg), and the ED95 was estimated at 5.308 µg/kg (95% CI: 5.065–6.496 µg/kg). Nicardipine effectively mitigated the hypertensive response caused by pituitrin without inducing significant tachycardia or hypotension.

**Conclusions:**

Nicardipine effectively controlled blood pressure after pituitrin injection during laparoscopic myomectomy, with ED50 and ED95 values established. This research highlights the potential utility of nicardipine in addressing hypertensive responses induced by pituitrin, particularly in clinical settings where pituitrin is routinely administered.

## Introduction

Uterine fibroids represent the most prevalent benign tumors of the female reproductive organs, with an incidence of 25-40% among women of childbearing age [1, 2]. Laparoscopic myomectomy has indeed emerged as the most common surgical approach, especially for individuals who desire to preserve their reproductive ability [3]. And the management of intraoperative bleeding is essential to the success of the procedure. In China, where synthetic vasopressin such as terlipressin is not commonly available, injecting pituitrin adjacent to uterine fibroids is a frequent alternative to reduce intraoperative blood loss during laparoscopic myomectomy [4]. Pituitrin is a bovine posterior pituitary extract that contains a mixture of oxytocin and vasopressin hormones, which can exert a hemostatic effect due to its similar clinical efficacy as synthetic vasopressin while being more cost-effective [5, 6]. Upon partial absorption into the bloodstream, pituitrin may elicit a range of hemodynamic responses, including transient hypotension and sustained hypertension [4], as well as arrhythmias such as bradycardia and myocardial ischemia [5, 7]. In severe cases, pituitrin injection can lead to cardiac arrest [4, 8].

The safe use of pituitrin in patients undergoing laparoscopic myomectomy and the maintenance of cardiovascular reactions within acceptable limits remain concerns for both anesthesiologists and surgeons. Considering the potential cardiovascular effects of pituitrin in the context of laparoscopic myomectomy and the need for effective management of intraoperative bleeding, exploring alternative medications that can mitigate pituitrin-induced hemodynamic responses becomes imperative. Dihydropyridine Ca^2+^ channel blockers are known to have potent coronary vasodilatory effects and have been widely used in patients with vasospastic angina [9]. Nicardipine, as a classic medication among dihydropyridine Ca^2+^ channel blockers, has become a first-line treatment for acute hypertension because of its long half-life, rapid onset of action, ease of use, and low price [10–12]. Additionally, nicardipine’s antihypertensive effect can reflexively elevate heart rate while increasing cardiac output, with minimal impact on the heart. Nicardipine also enhances coronary blood flow and improves myocardial oxygen supply [11]. Furthermore, research indicates that the time required for the nicardipine to achieve optimal blood pressure control is shorter than that of nitroglycerin, suggesting that nicardipine is more effective than nitroglycerin in managing acute hypertension [13]. Therefore, nicardipine holds promise in ameliorating the adverse reactions associated with pituitrin-induced hypertension and bradycardia. However, the ideal dosage of nicardipine to mitigate the impact of pituitrin-induced cardiovascular responses has not yet been thoroughly investigated. This study aims to explore nicardipine’s median effective dose (ED50) and 95% effective dose (ED95) of for treating pituitary-induced hypertension through a up-and-down sequential trial.

## Methods

### Study population

This study has received ethical approval from the Ethics Committee of Shanghai Changning Maternity and Infant Health Hospital (Ethics No.: CNFBLLKT-2021-008) and is registered in the China Clinical Registry (Registration No.: ChiCTR2100051320, first registration date: 20/09/2021). Informed consent has been obtained from patients. Inclusion criteria encompass patients who were scheduled for elective laparoscopic hysterectomy for uterine fibroid removal and met the following conditions: ASA classification I or II, aged between 18 and 55 years, and have a BMI ranging from 18 to 29 kg/m², and patients who achieve hypertension 5 min after injection of pituitrin. Patients will be excluded if they have communication difficulties, significant diseases affecting vital organs or systems such as ischemic heart disease, congestive heart failure, uncontrolled hypertension or diabetes, renal disease, or obesity (BMI > 30 kg/m²). Individuals with five or more uterine fibroids, or a largest fibroid diameter exceeding 10 cm, severe intra-abdominal adhesions, known allergy to bovine products, or those who refuse to participate in the trial.

### Study design

This study utilized a Dixon’s up-and-down method to determine the effective dose of nicardipine for treating intraoperative hypertension in patients undergoing surgery [14, 15]. The study followed a sequential design, where each patient’s treatment dose depended on the response of the previous patient. The initial dose of nicardipine was set as 6 µg/kg in the first round, which was based on previously published literature [16] and on experiences in our center. And a constant interval between adjacent doses was set as 0.5 µg/kg [14, 17]. The subsequent patient’s nicardipine dosage depended on the outcome of the preceding patient. If the previous patient’s blood pressure was successfully lowered, the dosage of nicardipine would be decreased by 0.5 µg/kg; conversely, it would be increased by 0.5 µg/kg.

### Patient monitoring

All patients underwent a routine fasting period of at least 8 h and a drinking ban of at least 2 h. Patients were closely monitored throughout the entire procedure after entering the operating room. Electrocardiography (ECG), invasive arterial blood pressure, heart rate (HR), peripheral oxygen saturation (SpO_2_), and bispectral index (BIS) values were recorded. Baseline blood pressure was measured in a calm state before anesthesia administration in the ward [18, 19].

### Anesthesia induction and maintenance

Anesthesia was induced using total intravenous anesthesia (TIVA) with the following medications: sufentanil (0.2 µg/kg), propofol via target-controlled infusion (TCI) at a target plasma concentration of 6 µg/ml, and cisatracurium (0.15 mg/kg). Tracheal intubation was performed after 3 min of assisted breathing. Mechanical ventilation was initiated in pressure-controlled ventilation with volume guarantee (PCV-VG) mode, maintaining a target tidal volume (VT) of 6–8 ml/kg based on ideal body weight. The respiratory rate (RR) was set at 10–14 breaths/min, and the partial pressure of end-tidal carbon dioxide (EtCO_2_) was maintained between 35 and 45 mmHg.

During the maintenance phase of anesthesia, propofol via TCI (target plasma concentration of 3–5 µg/ml) were administered to maintain anesthesia depth by keeping bispectral index (BIS) values between 40 and 60 throughout the operation. The infusion rate (target plasma concentration of 3–6 ng/ml) of remifentanil was modified to keep the surgical pleth index within the range of approximately 30 to 50 [20, 21] throughout the procedure. Cisatracurium (0.03 mg/kg) was administered every 20 to 30 min to sustain muscle relaxation.

### Surgical procedure and nicardipine administration

The patient was placed in the Trendelenburg position, with the feet elevated and head lowered. Pneumoperitoneum pressure was maintained at 8–10 mmHg. Under laparoscopic view, a puncture needle was inserted into the myometrium surrounding the fibroids. If no blood was observed upon aspiration before fibroid removal, a mixture of posterior pituitrin (6U, batch number: 230,101; Hongye Manufacturing Industry, Anhui, China) diluted with 0.9% normal saline to a total volume of 10 mL was injected. The concentration of the nicardipine (batch number: 2242P01; Daiichi Sankyo Company Limited, Tokyo, Japanese) solution was prepared by mixing 10 ml of nicardipine injection (10 ml: 10 mg) with 240 ml of saline, resulting in a concentration of 40 µg/ml. The dosage of nicardipine administered was based on the patient’s body weight and determined using the modified Dixon’s up-and-down method. If the blood pressure increased by more than 20% from the baseline following the injection of pituitrin, nicardipine was administered intravenously starting from 6 µg/kg.

### Data collection and processing

Following the administration of nicardipine, continuous monitoring of invasive blood pressure and heart rate was conducted, with recordings taken every minute for the initial 10 min and subsequently at 10-minute intervals for the following 20 min. The specific time points were designated as follows: T1 (1 min), T2 (2 min), T3 (3 min), T4 (4 min), T5 (5 min), T6 (6 min), T7 (7 min), T8 (8 min), T9 (9 min), T10 (10 min), T20 (20 min). Any occurrence of adverse reactions, including hypertension, hypotension, bradycardia, and tachycardia, within the 30-minute monitoring period was meticulously documented. The standard for diagnosing hypertension is a 20% increase in systolic blood pressure (SBP) compared to baseline. Hypotension is diagnosed with a ≥ 20% decrease in SBP from baseline. Bradycardia is identified with a ≥ 20% decrease in heart rate (HR) from baseline. Tachycardia is determined with a ≥ 20% increase in HR from baseline.

### Stopping rules

The stopping rules in this study were crucial to ensure the safety and efficiency of the nicardipine dosage determination for treating pituitary-induced hypertension during laparoscopic myomectomy.

Effective Case Definition: An effective case was defined as a scenario where the patient’s blood pressure returns to the target range (within ± 20% of baseline) within 120 s after the intravenous injection of nicardipine.

Rescue Treatment Protocol: If the patient’s blood pressure did not return to the target range within the expected time, rescued treatment with an additional dose of nicardipine may be administered as per the protocol. The termination of nicardipine and transition to other blood pressure-lowering medications will be at the discretion of the anesthesiologist.

Unsuccessful Case Criteria:

- Blood Pressure Not Controlled: If the blood pressure does not return to the target range within 120 s after the nicardipine injection, the case will be considered unsuccessful.
- Exceeding Maximum Nicardipine Dose: If the total amount of nicardipine administered to a patient exceeds 30 µg/kg, it will be labeled as an unsuccessful case since this exceeds the maximum allowable dose for a single bolus injection.
- Exceeding Pituitary Injection Dose: If the intraoperative bolus dose of the posterior pituitary injection exceeds 6U, the trial will be terminated, as this could have implications for the study’s objectives.
- Complications or Adverse Events: If any complications resulting from general anesthesia or medications occur, such as allergies, airway spasms, or pulmonary embolisms, the trial will be terminated for the safety of the patient.

### Statistics and sample size

The study followed a Dixon’s up-and-down sequential allocation trial design to determine the effective dose of nicardipine for treating intraoperative hypertension in patients undergoing laparoscopic myomectomy after intramyometrial injection of pituitrin.

Given the nature of the trial and drawing from previous studies, an estimated sample size of 20–40 patients was deemed appropriate for this study, aligning with the guidelines for the Dixon’s up-and-down method [14, 15, 17]. This sample size was chosen to ensure that there were a sufficient number of independent pairs of failure/success, which is necessary to reliably estimate the effective dose (ED50) and the ED95 of nicardipine for treating intraoperative hypertension after intramyometrial injection of pituitrin.

### Statistical analysis

Statistical analyses were conducted using IBM SPSS Statistics for Windows version 22 (IBM Corp., Armonk, NY, USA) and R statistical software version 3.4.0 (R Foundation for Statistical Computing, Vienna, Austria; http://www.R-project.org/). Descriptive statistics were used to summarize patient demographics and relevant characteristics. To determine the ED50 and ED95 of nicardipine for successful blood pressure control after pituitrin injection, probit regression was employed [22]. The 95% confidence intervals (CI) for probit regression were estimated using the Maximum Likelihood Estimation (MLE) method. Furthermore, paired t-tests or the Wilcoxon signed-rank test were conducted to compare blood pressure and heart rate at the time of nicardipine intravenous injection with values at other time points during the observation period. A significance level of 0.05 was utilized to determine statistical significance in all analyses.

## Results

**Fig. 1:**
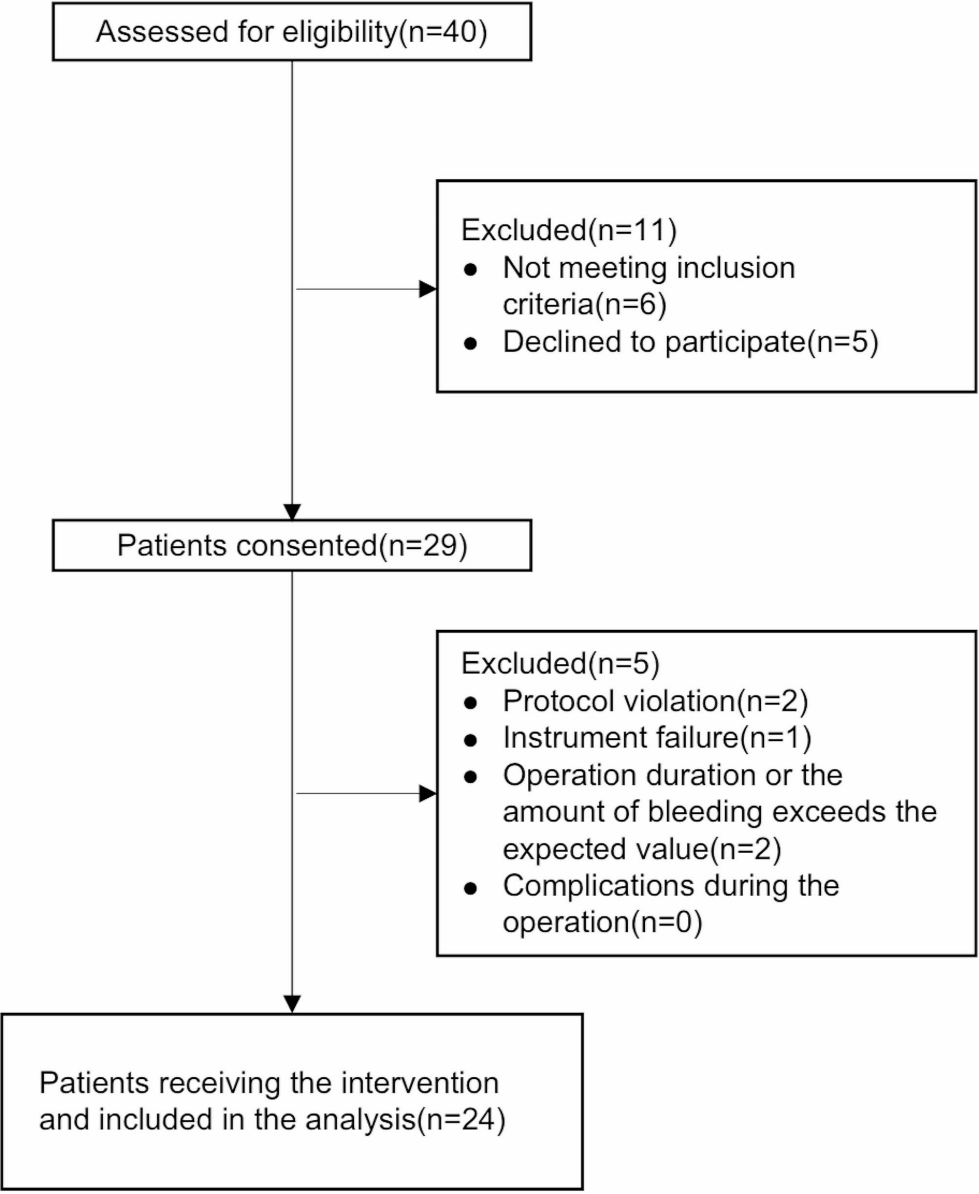
Flow-chart of the study

The flowchart illustrating the up-and-down sequential allocation study was depicted in Fig. 1.

Out of the initially assessed 40 participants, 11 were excluded for various reasons: 6 did not meet the inclusion criteria, 5 declined to participate, 2 had protocol violations, and 1 experienced instrument failure. An additional 2 participants were excluded due to unexpected operation duration or bleeding. No operation-related complications were observed. Consequently, 24 eligible participants received the intervention and were included in the analysis. The demographic data of patients in both the effective and ineffective groups are presented in Table 1. No significant differences were observed between the effective and ineffective groups in terms of age, BMI, Baseline BP, Baseline HR, and Number of fibroids (*P* > 0.05).

**Table 1:** Demographic data and patient characteristics

Group	Number of Cases(n)	Age (yr)	BMI (kg/m^2^)	Baseline SBP (mmHg)	Baseline HR (mmHg)	Number of Fibroids(n)
Effective	13	39.9 ± 5.5	24.8 ± 1.9	118.8 ± 6.5	69.4 ± 3.1	2(2–3)
Ineffective	11	35.9 ± 6.1	24.8 ± 1.7	115.6 ± 5.8	70.4 ± 6.4	2(2–3)

Values are expressed as mean ± SD, median(range), or number of patients. There were no significant differences observed between the groups in terms of age, BMI, Baseline BP, Baseline HR and Number of Fibroids (*P* > 0.05). BMI, body mass index; SBP, systolic blood pressure; HR, heart rate

**Fig. 2:**
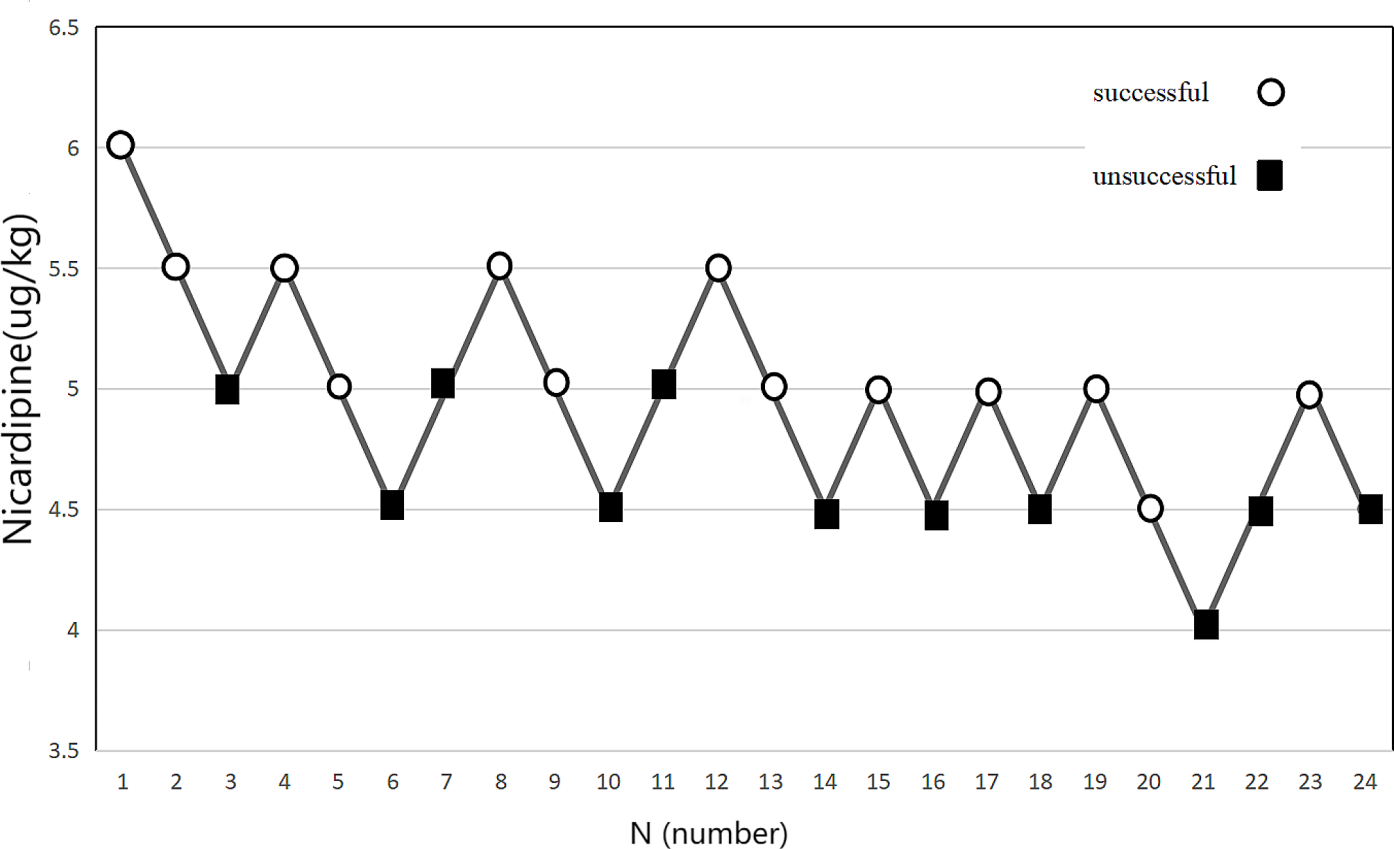
The sequential trial utilizing nicardipine to alleviate hypertension induced by intramyometrial injection of pituitrin

Sequential patient response to nicardipine injection with “up-and-down” method is shown in Fig. 2.

**Fig. 3:**
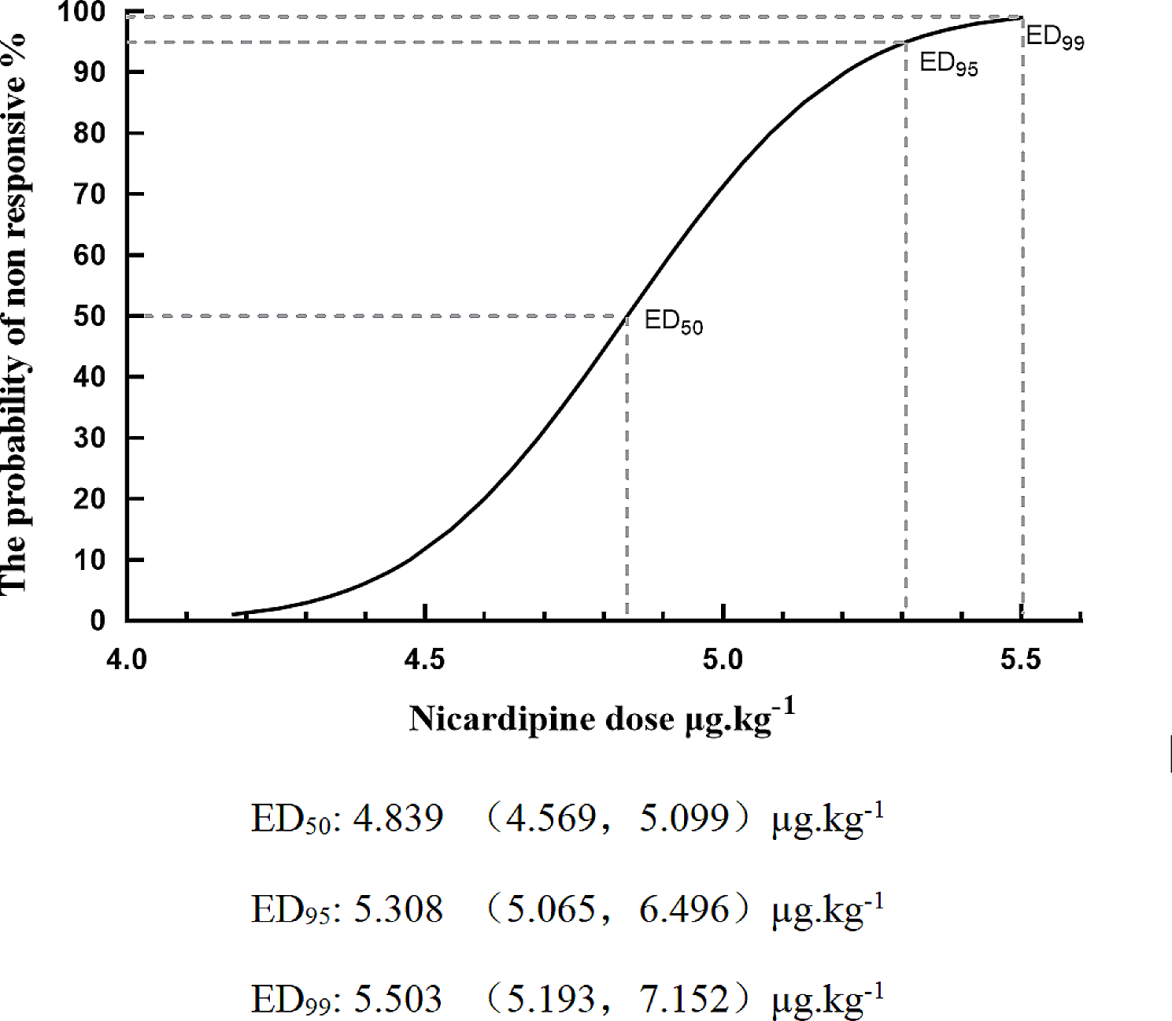
Dose-response curve

The dose–response curve of nicardipine for treating pituitrin-induced hypertension during surgeries is presented in Fig. 3.

The ED50 and ED95 values were 4.839 µg/kg (95%CI: 4.569–5.099 µg/kg) and 5.308 µg/kg (95%CI: 5.065–6.496 µg/kg), respectively. Table 2 presents the nicardipine doses and the corresponding percentages of patients with successfully controlled blood pressure.

**Table 2:** Nicardipine doses and percentages of patients with controlled blood pressure concentration of nicardipine(µg/kg)

Concentration of Nicardipine (µg/kg)	Success Rate (%/n)
6	100%(1/1)
5.5	100%(4/4)
5	70%(7/10)
4.5	12.5%(1/8)
4	0%(0/1)

Success case definition: An success case is defined as a scenario where the patient’s blood pressure returns to the target range (within ± 20% of baseline) within 120 s after the intravenous injection of nicardipine

**Fig. 4:**
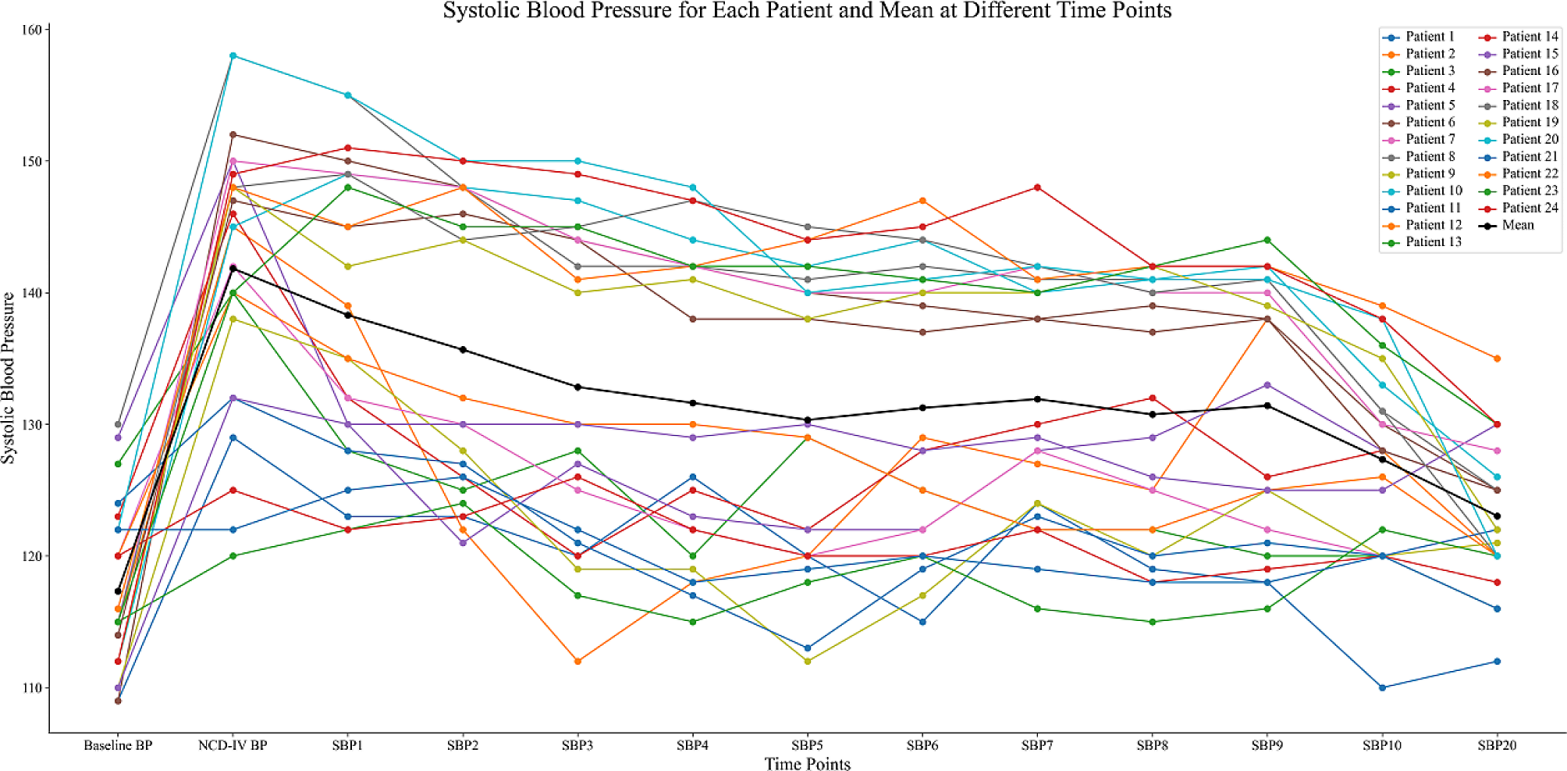
The variations of SBP within 20 min after injection of pituitrin

**Fig. 5:**
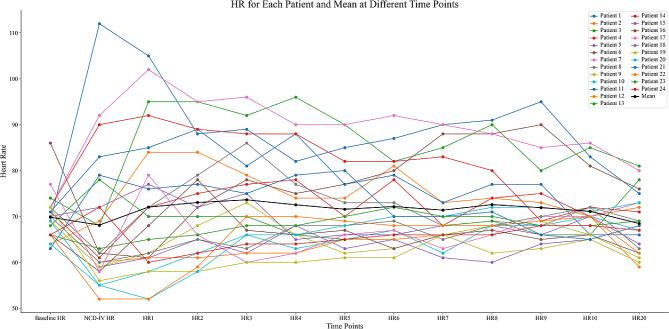
The variations of HR within 20 min after injection of pituitrin

The Variations of SBP and HR within 20 min after injection of pituitrin are presented in Figs. 4 and 5.

Analysis showed that SBP over time was significantly different within each group (*P* < 0.001). Compared with time point at the start of intravenous injection of nicardipine(NCD-IV BP), SBP at T1 (1 min after nicardipine injection) was decreased significantly (*P* < 0.05), and reached the lowest at T5, then keep a stable state.

## Discussion

Our study sheds light on the potential use of nicardipine in managing pituitrin-induced hypertension during laparoscopic myomectomy, a procedure aimed at reducing intraoperative bleeding. Pituitrin’s cardiovascular side effects, including hypertension and arrhythmias, necessitate an effective and safe countermeasure, and nicardipine, a dihydropyridine calcium channel blocker, emerges as a promising solution.

Our study established the ED50 and ED95 of nicardipine for managing pituitrin-induced hypertension during laparoscopic myomectomy, providing essential guidance for anesthesiologists and surgeons when pituitrin administration is required. With an ED50 of 4.839 µg/kg and an ED95 of 5.308 µg/kg, we offer a quantitative framework for determining the appropriate nicardipine dosage, ensuring both effectiveness and safety while minimizing potential side effects.

These findings hold particular relevance in clinical settings where pituitrin is routinely administered, such as in China. Nicardipine, known for its safety profile, rapid onset of action, and the ability to maintain heart rate and cardiac output, proves to be an ideal choice for managing the cardiovascular responses induced by pituitrin.

Nicardipine has shown effectiveness and safety in managing acute hypertension following various conditions, including post-subarachnoid hemorrhage, severe antepartum hypertension, hypertensive emergencies [23]. Nicardipine is also a versatile medication used to maintain intraoperative hemostability, managing conditions like tracheal intubation or extubation-induced hypertension, chronic hypertension, and inducing controlled hypotension for specific surgical procedures [11]. However, the ideal dosage of nicardipine for managing pituitrin-induced hypertension remains uncertain, which is why we initiated this trial.

This study demonstrated that the ED50 of nicardipine in attenuating pituitrin-induced hypertension during laparoscopic myomectomy in patients was determined to be 4.839 µg/kg (95% CI: 4.569–5.099 µg/kg) using a sequential method for single injection. Furthermore, the ED95 was estimated to be 5.308 µg/kg (95% CI: 5.065–6.496 µg/kg). These findings provide quantitative support for the inhibitory impact of nicardipine on the pressor response to pituitrin in this clinical context.

One research, using Dixon’s up and down sequential allocation, aimed to determine nicardipine’s ED50 for mitigating hypertensive response during tracheal intubation induction [24]. The results showed that the ED50 values for nicardipine in three groups (thiopental, propofol, and etomidate) were 18.0 µg/kg, 6.2 µg/kg, and 16.7 µg/kg, respectively. Our study results demonstrated that the ED95 for intravenous nicardipine in treating pituitrin-induced hypertension is approximately 5.308 µg/kg with a 95% confidence level. Notably, the propofol group exhibited the lowest nicardipine requirement, which aligns closely with our research findings.

The hemodynamic response triggered by pituitrin follows a biphasic pattern, primarily influenced by its constituents—vasopressin and oxytocin. Vasopressin binds to V1 receptors, prompting contraction of vascular smooth muscles, thus raising blood pressure and inducing peripheral vasoconstriction [4, 25, 26]. On the other hand, oxytocin receptors, present on endothelial cells, promote intracellular calcium ion increase, augmenting endothelial nitric oxide synthase activity and consequent vasodilation [27]. The interplay of vasopressin, with a longer half-life, and oxytocin concentration appears pivotal, often resulting in sustained elevation of blood pressure compared to the initial decrease [28]. In a recent study, the dose-response of nitroglycerin in preventing pituitrin-induced hypertension during laparoscopic myomectomy was investigated [29]. The results indicated that nitroglycerin effectively prevented pituitrin-induced hypertension, and specific doses were associated with a significant reduction in hypertension incidence. However, prophylactic nitroglycerin used prior to pituitrin-induced hypertension may lead to higher incidence of hypotension. Therefore, nicardipine is milder than nitroglycerin, and previous study even suggest it is suitable for patients with hypertensive acute heart failure syndrome [30]. Our study was designed to mitigate the hypertensive response that typically occurs approximately 1 min after the intramyometrial injection of pituitrin [4]. This approach intentionally excludes patients who may experience oxytocin-induced hypotension, contributing to the safety of pituitrin administration. In addition, previous studies have shown that the total dose of pituitrin should not exceed 6 units, otherwise it can lead to more severe multimodal fermentation [1–4]. Adhering to meeting the hemostatic needs of surgeons to the maximum extent possible, we have used a maximum of 6U of posterior pituitary hormone.

Nicardipine, a water-soluble calcium channel antagonist, primarily exerts vasodilatory effects. In situations demanding rapid blood pressure control, intravenous nicardipine demonstrates a relatively swift onset and offset of action. The pharmacokinetics of nicardipine in patients with mild to moderate hypertension follow a linear pattern [31]. A large mount of studies investigate nicardipine’s role in mitigating the intraoperative stress response. Intravenous nicardipine effectively controls acute intraoperative blood pressure across a spectrum of surgical procedures, encompassing cardiovascular, neurovascular, and abdominal surgeries [11]. It is particularly beneficial in intentional blood pressure reduction during surgeries where achieving hemostasis poses challenges, such as procedures involving the hip or spine [32]. Moreover, multiple studies demonstrate that intravenous nicardipine attenuates the hypertensive response without inducing tachycardia in anesthetized individuals following laryngoscopy and tracheal intubation [33]. A well-designed, sizable study affirms nicardipine’s ability to prevent cerebral vasospasm in patients with recent aneurysmal subarachnoid hemorrhage [34], consolidating evidence for its effectiveness in short-term hypertension treatment. This study presents the inaugural investigation into the median effective dose of nicardipine for averting pituitrin-induced hypertension during laparoscopic myomectomy.

Research indicates that intravenous nicardipine is as effective as, and in some cases, even superior to other medications used for emergency blood pressure reduction, such as nitroglycerin, in managing preoperative hypertension during surgery. Nicardipine offers specific advantages, including a stable dose-response effect and less hypotension and tachycardia [35]. Additionally, another study demonstrated that nicardipine has a higher likelihood of achieving the physician-specified SBP (systolic blood pressure) target range within 30 min compared to labetalol [36].

In our study, we utilized a sequential trial approach to determine the optimal dosage of nicardipine for managing pituitrin-induced hypertension during laparoscopic myomectomy. The adaptive nature of a sequential trial allowed us to efficiently evaluate medication responses within a relatively short time frame, which is especially crucial in a clinical context where rapid intervention is required to address hypertensive episodes induced by pituitrin [37].

The dose-response relationship is a fundamental concept that guided our investigation. Our aim was to find the most effective dosage of nicardipine, and the dose-response relationship, with a focus on quantal response, precisely reflects how different doses of the drug interact with the body. Understanding this relationship is critical for optimizing the therapeutic effect of nicardipine while minimizing any potential adverse outcomes [38].

Limitations of this study include: first, we administered a single bolus of nicardipine without combining it with pump injections. It is possible that the antihypertensive effect of the drug gradually weakens several minutes after bolus injection. The combination of bolus and pump infusion requires further research. Second, the main drawback of the sequential trial is that the study results tend to be conservative, potentially delaying the clinical implementation for potentially beneficial procedures. Moreover, a larger sample size may be necessary for rare events. The last, this study is only suitable for nicardipine ED50 and ED95 under total intravenous anesthesia. Therefore, this investigation is instructive as a pilot study for preventing pituitrin-induced cardiovascular adverse reactions in patients undergoing laparoscopic myomectomy with nicardipine.

In conclusion, nicardipine effectively inhibits pituitrin (6U)-induced cardiovascular adverse reactions in patients undergoing laparoscopic myomectomy, with an ED50 of 4.839 (95% confidence interval: 4.569–5.099) µg/kg and an ED95 of 5.308 (95% confidence interval: 5.065–6.496) µg/kg.

## Data Availability

The data sets used and/or analyzed during the current study are available from the corresponding author on reasonable request.
